# A new era in palaeomicrobiology: prospects for ancient dental calculus as a long-term record of the human oral microbiome

**DOI:** 10.1098/rstb.2013.0376

**Published:** 2015-01-19

**Authors:** Christina Warinner, Camilla Speller, Matthew J. Collins

**Affiliations:** 1 Department of Anthropology, University of Oklahoma, Norman, OK, USA; 2 Department of Archaeology, University of York, York, UK

**Keywords:** palaeomicrobiology, ancient DNA, oral microbiome, dental calculus, metagenomics, metaproteomics

## Abstract

The field of palaeomicrobiology is dramatically expanding thanks to recent advances in high-throughput biomolecular sequencing, which allows unprecedented access to the evolutionary history and ecology of human-associated and environmental microbes. Recently, human dental calculus has been shown to be an abundant, nearly ubiquitous, and long-term reservoir of the ancient oral microbiome, preserving not only microbial and host biomolecules but also dietary and environmental debris. Modern investigations of native human microbiota have demonstrated that the human microbiome plays a central role in health and chronic disease, raising questions about changes in microbial ecology, diversity and function through time. This paper explores the current state of ancient oral microbiome research and discusses successful applications, methodological challenges and future possibilities in elucidating the intimate evolutionary relationship between humans and their microbes.

## 1. Introduction

Palaeomicrobiology is an important and growing area of archaeological [1] and microbiological [2] research. It has developed in parallel with palaeoenvironmental studies exploring microbial activity in deep subsurface environments such as oil [3], the deep sea [4] and permafrost [5–9], all of which have revealed that microbial DNA can persist in ancient deposits. With respect to humans, the study of ancient microorganisms has the potential to reconstruct human migration and interaction networks [10], and to identify the origins, causes and evolution of specific infectious diseases [11–14]. Historically, however, the promise of palaeomicrobiology has been tempered by the uneven quality of research; the field has been plagued, so to speak, by high profile controversies [15–19], and bold claims made on the basis of modest, incomplete, or problematic evidence have been met with scepticism, doubt or outright rejection by the broader ancient DNA community [20,21]. At issue is the fact that we live in a world dominated by microorganisms, both in absolute numbers and in species diversity [22–24], and palaeomicrobiology studies have often failed to account adequately for issues of contamination, authenticity and sequence specificity in their experimental design. In a review paper as recently as 2005, the study of ancient bacterial DNA could be accurately summarized as ‘the microbial problem’, with few prospects for resolution [25].

However, recent improvements in contamination control [26], laboratory workflow design [27,28] and the emergence of powerful new sequencing technologies [29,30] and bioinformatics tools [31–34] are dramatically altering both the practice and potential of ancient microbial research. High-throughput next generation sequencing (NGS) presents a solution to many of the challenges surrounding conventional molecular methods of pathogen identification, and it additionally expands scientific inquiry beyond pathogen presence/absence to questions of pathogen evolution, genetic mutation, genome rearrangement and horizontal gene transfer.

A major recent advancement in palaeomicrobial research has been the discovery that dental calculus acts as a long-term reservoir of high-quality biomolecules from human-associated microorganisms [35–39]. While this substrate was previously recognized to contain calcified bacterial cells [40] and dietary microfossils [41–44], and was later shown to preserve host mitochondrial DNA [36] and biomolecules from a few select bacterial species [36,37], the application of high-throughput sequencing has now allowed the recovery of entire ancient microbial communities [35,39], also known as the native human microbiota or ‘microbiome’ [45]. This enables palaeomicrobiology to move beyond Koch's influential postulate of ‘one pathogen—one disease’ to investigate the full suite of ‘commensal, symbiotic and pathogenic microorganisms’ that contribute to human health and disease both today and in the past [45,46].

Emerging out of technological innovations developed during the race to sequence the human genome, NGS is now being widely mobilized to investigate the structure and function of the human microbiome in populations around the world. Projects such as the National Institutes of Health's Human Microbiome Project (HMP) in the United States and the Metagenomics of the Human Intestinal Tract (MetaHIT) project in Europe have revealed that the human oral, gut, skin and uritogenital microbiota play critical roles in promoting and maintaining human health. Disruption of these microbiomes leads to dysbiosis, a detrimental relationship between microbiota and host that is linked to illnesses as diverse as obesity and type II diabetes [47,48], periodontal disease and dental decay [49,50], atherosclerosis and endocarditis [51,52], eczema [53], vaginosis [54] and inflammatory bowel disease [55], among others.

Determining effective methods for treating disturbed microbiomes is of great medical interest and requires a nuanced understanding of what constitutes a healthy microbiome. At present, however, remarkably little is known about the diversity, variation and evolution of the human microbiome, both today and in the past. Nor is it well understood how our microbiome health is linked to our genetic background, cultural practices and environment. Accessing ancient microbiomes through archaeological data presents a unique approach for investigating the ecology and evolution of the oral microbiome prior to our post-industrial lifestyle, globalized food chain and antibiotic use. Focusing on dental calculus, this paper will discuss the potential of ancient microbiome research, as well as current methodological challenges.

## 2. The oral microbiome

**Figure 1.**
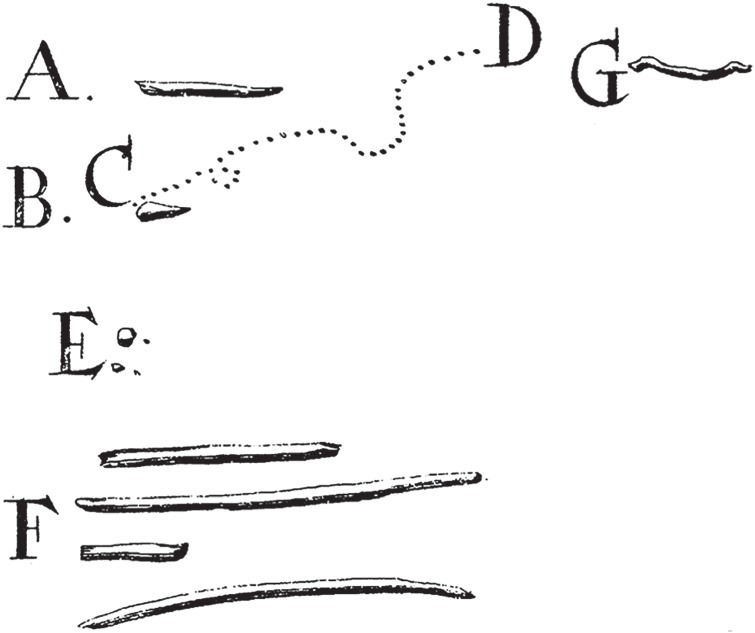
Early illustration of dental plaque bacteria by Antoni van Leeuwenhoek, 1683/1684. Illustrated bacteria include (A) a rod-shaped motile bacterium, (B) another motile bacterium moving from points (C) to (D), (E) cocci, (F) fusiform bacteria and (G) a spirochaete. Adapted from [57].

The oral microbiome, and dental plaque in particular, holds a special place in the history of microbiology [56]. The first undisputed description of bacteria appears in a letter written by Antoni van Leeuwenhoek to the Royal Society of London in 1683 in which he describes ‘very many small living Animals, which moved themselves very extravagantly’ within his dental plaque [57]. Familiar oral bacterial forms can be found among his illustrations, including cocci, fusiform bacteria and spirochaetes (figure 1) [58]. Attempting in vain to count them, he noted, ‘The number of these animals in the scurf of mans [sic] Teeth, are so many that I believe they exceed the number of Men in a kingdom.’ [57].

Van Leeuwenhoek's analogy is, if anything, understated. The average healthy person carries on the surface of their teeth nearly as many bacteria as there are humans on the Earth [59], and every day each of us swallows an average of 80 billion bacteria in our saliva [60]. Within the oral cavity, the teeth are like mountains, saliva like the high seas and in between are the forests of the tongue, the savannahs of the mucosa and the dark swamps of dental plaque. Populated by fusobacteria and streptococci and treponemes, rather than trees and birds and fish, these complex oral landscapes support an incredible diversity of microbial life. The human oral cavity is thus more than a kingdom, it is an entire world unto itself.

The oral microbiome is the second largest human-associated microbial community, after the gut, and oral microbes exhibit an astounding diversity of predicted protein functions compared with other body sites [61]. The oral cavity can be divided into several distinct oral habitats, each with its own characteristic microbial composition. Even sites with frequent contact, such as the hard palate and the tongue, persist in maintaining different microbial ecologies [62]. Despite these differences, however, the greatest distinction is observed between bacterial communities inhabiting shedding soft tissue surfaces (e.g. buccal mucosa, keratinized gingiva, tongue dorsum, hard palate, tonsils and throat) and non-shedding hard tissue surfaces (dental plaque). Saliva, another oral habitat, is a complex biofluid that contains bacteria from both soft and hard surfaces, but its microbial community most closely resembles those of the soft tissues. The hard tissues of the teeth provide two microbial habitats, one above and one below the gingival margin, resulting in two distinctive plaque communities known as supragingival and subgingival plaque, respectively [63,64]. These two habitats differ in redox potential and nutrient sources, with supragingival plaque forming in a more aerobic environment fed by nutrients of primarily salivary origin and subgingival plaque forming in a mostly anaerobic environment fed by gingival crevicular fluid (GCF), an inflammatory exudate of the gingiva.

## 3. Dental calculus

Dental calculus (tartar, or calcified dental plaque) is a complex, mineralized bacterial biofilm formed on the surfaces of teeth, principally from dental plaque but also with additional contributions from saliva and GCF [65,66]. Dental calculus is found in all known human populations, past and present, and is nearly ubiquitous in adults without active dental hygiene [67,68]. Biofilm formation begins when salivary proteins deposit as a thin film on the surface of the teeth, forming the acquired enamel pellicle (AEP). During life, the AEP serves as the primary barrier and defensive layer between the calcium phosphate mineral of the enamel and bacterial and dietary acids [69]. Shortly after AEP formation, oral bacteria capable of hard surface adhesion, mostly Gram-positive viridans streptococci and *Actinomyces* species, begin colonizing the surface of the pellicle, followed by ordered waves of microbial succession, forming a complex, structured plaque [70–72] with a bacterial density of more than 200 million bacterial cells per milligram [72,73]. The plaque is held together by a glycocalyx matrix of bacterial extracellular polymeric substances (EPSs) that include exopolysaccharides and cell lysis and hydrolysis products, as well as extracellular DNA (eDNA) [73–75]. High molecular weight eDNA has been shown to play a role in initial biofilm formation [76], and in addition to serving a structural function, eDNA may also play a role in the horizontal transfer of antibiotic resistance and other genes within oral biofilms [77–79].

For reasons that are not fully understood [63,65,66,68,80], dental plaque undergoes periodic mineralization events to form dental calculus. Calcium phosphate ions from saliva and GCF precipitate within supragingival and subgingival dental plaque, respectively, first in the intercellular matrix and later within a portion of the bacterial cells. During this phase, the AEP also calcifies, and any irregularities or pits on the surface of the tooth are also infilled with crystals, further strengthening the attachment of the calculus to the tooth [63]. Dental calculus mineral is similar to that of bone and dentine and is composed of multiple calcium phosphates with different morphologies and stoichiometric compositions that change during biomineral maturation [65,66] to form a cement-like substrate with high physical hardness and adhesive strength [68].

The dominant phases of calcium phosphate in dental calculus are (in order of increasing crystallinity): brushite (B), octocalcium phosphate (OCP), whitlockite (TCP-b) and hydroxyapatite (HAP). DNA is known to bind strongly to calcium phosphate minerals [81], and mineral growth around and within oral bacterial cells may directly aid in nucleic acid survival [82]. During maturation, the crystallinity of dental calculus increases, with interior layers exhibiting more high-crystallinity phases (e.g. HAP) than exterior layers [66]. Nevertheless, all four phases are found together within mature dental calculus, and even within archaeological specimens [83]. After mineralization is complete, the process of plaque formation begins again and the cycle continues, resulting in an incremental and appositional growth of dental calculus deposits [80].

**Figure 2.**
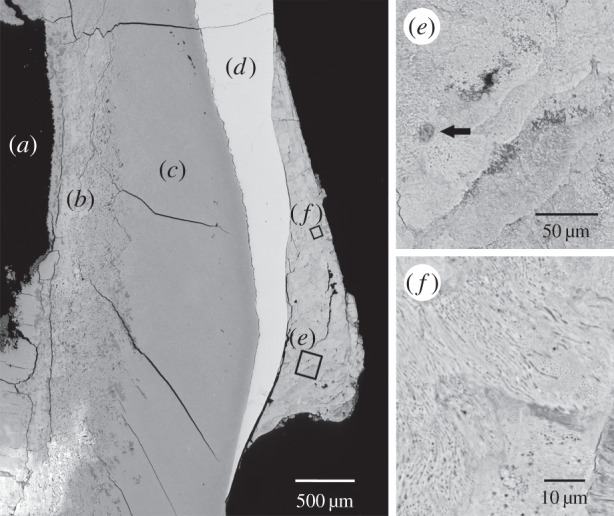
Backscattered scanning electron microscopy image of archaeological dental calculus *in situ* on the labial surface of a mandibular incisor. (*a*) Dental pulp cavity; (*b*) taphonomically altered dentine; (*c*) intact dentine; (*d*) enamel; (*e*) detail of dental calculus mineral layers and an *in situ* microfossil inclusion of biogenic silica (arrow); (*f*) detail of oral bacteria within dental calculus. The specimen shown is from Dalheim, Germany, and has been radiocarbon dated to 1079 ± 51 CE (calibrated) [39].

During this process of biomineral maturation, dietary microfossils (e.g. phytoliths, starch granules and pollen) may also become incorporated into dental calculus. Likewise, airborne and waterborne environmental pollutants, such as microcharcoal and sponge spicules, can become entrapped within the calcifying plaque, as can cooking and craft activity waste, such as groundstone grit and plant and animal fibres. The result of these processes is a mineralized bacterial biofilm that adheres to the surface of the tooth and contains a temporally ordered succession of diverse bacterial cells and environmental debris fossilized *in situ* (figure 2).

## 4. Dental calculus in archaeological research

There is growing recognition of the importance of archaeological dental calculus as a source of oral health and dietary information. Early studies of archaeological dental calculus can be traced back nearly a century [84], but it was not until the 1960s and 1970s that dental calculus began to receive serious treatment by archaeologists, dental anthropologists and dentists, who described its occurrence in both human [83,85–88] and faunal [41] assemblages and determined its mineral composition [83,88]. During the 1980s, dental calculus was documented in a range of archaeological populations [43,89–91], and systematic protocols were developed for recording dental calculus distribution and severity [92]. Throughout the 1980s and 1990s, interest in dental calculus continued to grow as its occurrence was observed to correlate at least in part with subsistence strategy [40,43,67,93,94], and by the mid-1990s dental calculus recording became a standard practice in the analysis of human remains [95].

In addition to macroscopic analysis, microscopic investigation of dental calculus also greatly advanced during the late 1980s and 1990s. Pioneering work by Dobney & Brothwell [40,43] revealed a great diversity of well-preserved microbial and dietary microfossils within the archaeological dental calculus of both humans and fauna. Building on this and other foundational work by Armitage [41], the early 1990s witnessed a dramatic growth in plant microfossil research focusing primarily on phytolith recovery from extinct primate [96], faunal [94,97] and human [44,94,98] dental calculus. In parallel, scanning electron microscopy (SEM) of human [37,93,94,99,100], archaic hominin [93,101,102] and extinct primate dental calculus [103] revealed the presence of well-preserved bacterial forms within dental calculus spanning time periods dating back to the Miocene (*ca* 9.3 Ma).

In the 1990s and 2000s, starch granule analysis of dental calculus made fundamental contributions to reconstructing the starchy components (e.g. roots, tubers, seeds) of human [42,104–106] and archaic hominin [107,108] diets, and both starch granule taphonomy [108,109] and dental calculus pyrolysis profiles [107] have additionally been used to infer past cooking practices. Dental calculus-based palaeodietary inference has also been attempted using trace element [110] and stable isotope [111,112] methods; however, the latter approach has received sharp criticism [113]. Moving beyond dietary analysis, observations of plant textile fibres within archaeological dental calculus also indicate that it is a potential source of information about past human craft activity and trade [114].

The first biomolecular investigation of dental calculus was conducted in 1996 and aimed to identify the oral pathogen *Streptococcus mutans* (a causative agent of dental caries) through immunohistochemical analysis [37]. In 2011, the preservation of bacterial DNA within dental calculus was confirmed by gold-labelled antibody transmission electron microscopy [38], and this was followed in 2012 by targeted PCR-based genetic approaches, which identified *S. mutans* and additional oral taxa, including *Fusobacterium nucleatum*, *Actinomyces naeslundii*, *Porphyromonas gingivalis* and *Streptococcus gordonii*, as well as human mitochondrial DNA [35,36]. With the application of NGS in 2013, Adler *et al.* [35] used 16S rRNA gene amplicons to demonstrate that dental calculus preserves an oral microbiome profile. They recovered microbiome data from individuals spanning the Mesolithic through to modern day, and investigated two phylum-level ecological shifts coinciding with the origins of agriculture and industrialization. Subsequently, Warinner *et al.* [39] performed a species-level taxonomic and protein functional characterization of ancient oral microbiomes in 2014 with the use of shotgun metagenomics and metaproteomics. This approach allowed a detailed analysis of ancient periodontal disease on the basis of bacterial virulence factors and host immune activity, genome reconstruction of the periodontal pathogen *Tannerella forsythia*, and identification of specific plant and animal dietary components. These studies provide a glimpse of the potential wealth of evolutionary, health and dietary information that dental calculus research can provide as more geographically and temporally diverse populations are investigated.

## 5. Advancing the field of ancient oral microbiome research

There is immense public interest in the emerging field of microbiomes and excitement about the extension of this research into the past. However, as with all emerging areas of research, there will be growing pains and methodological challenges to be faced and overcome. In the past, palaeomicrobiology studies were primarily challenged by too little data—insufficient DNA yields for sequencing, promising sequences that could not be replicated and incomplete datasets. Today, in the era of NGS and high-throughput mass spectrometry, the challenge is more likely to be too much data and how appropriately to manage, filter, assemble, authenticate and interpret the millions of sequences and spectra that make up current palaeomicrobial datasets [115,116]. In this section, we will examine current methodological challenges and opportunities relating to sampling, biomolecule extraction, microbiome characterization and contamination management in the emerging new field of dental calculus palaeomicrobiology.

### (a) Sample collection

The study of ancient microbiomes is still in its infancy, and there is much work to be done to optimize dental calculus sampling strategies and biomolecule extraction methods. At present, there is no consensus on optimal sampling strategies for dental calculus, either in terms of sample quantity or sampling location (e.g. dental quadrant or tooth type, labial/buccal versus lingual deposits). Ideally, supragingival and subgingival calculus should be collected and analysed separately, as they are known to have distinct aetiologies and different clinical significance [68]; however, in practice they are often difficult to distinguish in archaeological specimens. In the absence of soft tissue, it can be difficult to reconstruct the location of the former gingival margin, and archaeological subgingival calculus is not always darkened or discoloured, as it typically appears in living patients. Additionally, supragingival calculus may form on top of subgingival calculus as the alveolar margin recedes during the progression of periodontal disease. As a practical matter, sampling strategies are also often constrained by the teeth that are available in a given skeletal assemblage, as both ante- and post-mortem tooth losses are common. In many cases, pooling of calculus samples from multiple teeth may be the best method for obtaining representative data for comparison among individuals.

As with all destructive sampling techniques, the dentition should be photographed, and the location and severity of calculus should be documented prior to collection [43,95,117]. The additional sampling of associated dentine and/or bone may assist with characterizing the contamination burden of the burial environment. Because dentine and bone are typically sterile during life, bacteria recovered from these tissues represent highly local proxies for the post-mortem bacterial contamination that may be found in ancient microbiome samples [39]. Recently, electron microscopy of archaeological tooth sections revealed that environmental bacterial infiltration is greatest in cementum and in the dentine immediately surrounding the pulp cavity (figure 2). In addition, the lower portion of the tooth root in proximity to the root canal and apical foramen, which serves as the post-mortem entry point for environmental microbes into the pulp cavity, may show substantial taphonomic alteration [39]. These findings complicate recent suggestions to sample preferentially dental pulp, cementum and the tooth root tip for recovery of endogenous host DNA [15,118], as these sites appear to be the most taphonomically altered locations in archaeological teeth.

Finally, because of the wealth of microbial, health, environmental and dietary information potentially present within dental calculus, it is important to conduct the sampling and analysis of these substrates carefully and responsibly. To conserve material, unified protocols that can recover multiple types of information (e.g. DNA, proteins, microfossils and elemental/isotopic data) from the same starting material are urgently needed, and, as with all studies of ancient material, it is strongly recommended always to reserve a reasonable quantity of sample material for future analyses.

### (b) Biomolecule recovery

Throughout the 1990s and first decade of the 21st century, optimizing and maximizing DNA recovery from bone and dentine was a major focus of the ancient DNA community. To the best of our knowledge, only one study to date has compared the efficiency of different extraction protocols on archaeological dental calculus [39], and digestion buffer composition and extraction methods were found to impact DNA recovery yields by more than an order of magnitude. Similar variability in extraction efficiency has also been observed for modern microbiome samples, and minor changes in DNA extraction techniques have been found to impact recovery of specific taxa [119], an issue yet to be fully explored in dental calculus studies.

The amount of DNA preserved within some dental calculus samples is extraordinary, especially when compared with dentine (table 1). Comparing DNA yields from paired dental calculus and dentine samples, Warinner *et al.* [39] reported DNA yields as high as 437 ng mg^−1^ from dental calculus compared with 0.6 ng mg^−1^ from dentine of the same tooth, making dental calculus one of the richest known sources of ancient biomolecules in the archaeological record. However, DNA yields and downstream enzyme inhibition varied substantially depending on the digestion buffer and extraction method used, and attempts to remove inhibitory molecules resulted in substantial DNA loss. In the same study, Warinner *et al.* [39] also extracted proteins from dental calculus using a modified method originally developed for archaeological bone [120]. Although effective, enzyme inhibition during the trypsin digestion step reduced the efficiency of peptide generation. Removal of co-extracted inhibitory molecules, therefore, remains an obstacle in both metagenomic and metaproteomic dental calculus research.

**Table 1.** Comparative DNA yields between dental calculus and dentine. Normalized DNA yields are reported as nanogram DNA extracted per mg of tissue; DNA measurements determined using a Qubit fluorometer. n.d., not determined; asterisk (*) denotes mean yield of two extractions.

samples	dentine DNA yield (ng mg^−1^)	dental calculus DNA yield (ng mg^−1^)
Modern		
P2^a^	n.d.	83.4
POK1^b^	n.d.	346.0
POK2^b^	n.d.	313.5
Victorian		
FW283T^c^	23.1	13.4
Medieval		
G12^a^	0.5	44.8
B17^a^	0.3	437.2
B61^a^	0.3	5.0*
B78^a^	0.4	29.8
UK1^a^	n.d.	226.6*
Anglo-Saxon		
NEM093^c^	1.3	22.2
Roman Britain		
3DT21^c^	0.5	15.8
UK2^a^	n.d.	84.8

^a^Data from [39].

^b^DNA extractions performed using method A described in [39].

^c^DNA extractions performed using method A with silica modification described in [39].

### (c) Characterizing the ancient oral microbiome

A major challenge as we move forward in palaeomicrobiology will be to find optimal methods for characterizing ancient microbiomes, in terms of both taxonomic and functional profiles, that are compatible with modern datasets. At present, there are three primary approaches to characterizing the microbiome that have been applied to ancient samples: (i) amplicon sequencing, (ii) shotgun metagenomics, and (iii) shotgun proteomics.

#### (i) Amplicon sequencing

Amplicon sequencing is currently the standard in human microbiome characterization, as it is relatively inexpensive and is supported by a large body of comparative data in curated databases (e.g. RDP [121], SILVA [122] and Greengenes [123]) and established platforms for data management and analysis (e.g. QIIME [31] and Mothur [34]). Microbiome amplicon sequencing primarily focuses on one or more of the nine variable regions (V1–V9) of the 16S rRNA gene, a highly conserved ribosomal gene present in bacteria and archaea. Sequence divergence within the 16S rRNA gene variable regions is generally sufficient to distinguish bacterial taxa to the level of genus, and in some cases, species, and thus deep sequencing of 16S rRNA amplicons allows the taxonomic structure and diversity of a microbiome to be characterized. However, one challenge for an amplicon-based approach is the fact that many of the primer sets used to amplify 16S rRNA gene variable regions in ecological studies, such as 515F/806R [124] and 357F/926R [61], target regions greater than 300 bp in length and so exceed the DNA fragment lengths typical of ancient DNA. As a result, ancient DNA studies must rely on alternative, shorter primer sets with both altered primer-binding affinity and reduced taxonomic discriminating capacity. The third (V3) and sixth (V6) variable regions of the 16S rRNA gene are sufficiently short for ancient DNA studies (less than 200 bp) and have been successfully amplified from ancient dental calculus [35,39]. Both primer sets, however, may result in biased amplification of oral bacteria. For example, *in silico* primer analysis using PrimerProspector [125] predicts poor V3 primer binding affinity to spirochaetes, while V6 primers show poor binding affinity to TM7 phylum bacteria [39]. Bacterial frequency estimates from amplicon data are also complicated by the fact that many bacterial species have multiple copies of the 16S rRNA gene [126]. Finally, taxonomic dropout is also possible if DNA preservation is poor and amplification efficiency is low. Each of these factors must be taken into account when interpreting and comparing 16S rRNA amplicon sequencing data.

#### (ii) Shotgun metagenomics

Although not yet routine, shotgun metagenomics is gaining popularity as a community characterization approach. Rather than amplifying and sequencing a single gene or target region, as in amplicon sequencing, shotgun metagenomics randomly amplifies and sequences a subset of the total DNA in a sample. In this way, the entire biotic content of a sample (bacteria, archaea, eukarya and viruses) can be analysed at once, something that is not possible with amplicon sequencing because of the absence of conserved regions across all domains. Additionally, shotgun metagenomics does not suffer from issues of primer bias, although GC bias may still be a factor depending on the DNA polymerase used to prepare the sequencing library [127].

Shotgun metagenomics is potentially the most informative genetic approach to microbiome characterization, but it is also the most difficult to analyse and interpret [33,128]. Shotgun metagenomic datasets are massive (on the order of billions of nucleotides), depth of coverage is typically low, and there are few established analysis pipelines. Determining ‘who's there’ in a shotgun metagenomics dataset is far from straightforward, and may require using computationally intensive BLAST search algorithms, either before or after *de novo* contig assembly of sequencing reads, followed by labour intensive quality checking [39]. Recently, tools such as MEGAN [129], MG-RAST [130], mBLAST [131] and MetaPhlAn [64] have attempted to simplify the bioinformatic complexity of answering this question; however, each tool has its own limitations and biases with respect to specificity and inclusivity. For example, because MEGAN relies on only the top 100 BLAST hits for taxonomic assignment, it is susceptible to database bias and has a tendency incorrectly to assign conserved sequences to well-studied organisms with many NCBI entries, such as *Mycobacterium tuberculosis*. MetaPhlAn aims to provide quantitative assessments of metagenomic data, but its reliance on a restricted genomic database means that not all microbiome members are detected. This can lead to underreporting of some important taxa, such as the periodontal pathogen *T. forsythia*, which is not detectable using MetaPhlAn v. 1.7.7. For each of these tools, a detailed understanding of how they work, their biases and their limitations is essential in order to avoid misinterpretation of results. Another challenge of shotgun metagenomics analysis is that amplicon sequencing and shotgun metagenomics may reconstruct different bacterial communities [132], and recent gut microbiome analyses have found that shotgun metagenomic approaches yielded lower species diversity estimates than those based on amplicon sequencing of the 16S rRNA gene [133], suggesting biases of diversity underestimation and the need for improved computational analyses.

However, the true advantage of shotgun metagenomics is that it generates whole genome sequencing data; thus, downstream analyses are not limited to simple questions of taxonomy or phylogeny, but rather can extend to complex questions relating to gene content and genomic functional potential. The pairing of shotgun metagenomics with target enrichment has already allowed for the successful reconstruction of ancient *M. tuberculosis* [13], *Mycobacterium leprae* [14] and *Yersinia pestis* [12] genomes from ancient bone and dentine. Shotgun metagenomics can also be used to reconstruct genomes from the microbiome without enrichment, as has been demonstrated for *T. forsythia* [39]. Additionally, working with modern dental plaque samples, Liu *et al.* [134] recently reconstructed a partial genome for an uncultured TM7 bacterium without the aid of a reference genome. This achievement is significant for two reasons: first, it suggests that shotgun metagenomics may offer a potential solution to the problem that most microbes cannot be cultured in a laboratory; and second, it opens the door for future studies aimed at recovering extinct microbial genomes for which no reference genomes exist.

Microbiome community complexity, however, remains a significant challenge, and genome reconstruction is largely limited to highly abundant taxa. Moreover, strain genomic variability is typically high within microbiomes due to elevated levels of horizontal gene transfer and recombination, and thus a single reference genome is rarely sufficient to characterize a species. For example, the protein coding sequences (CDSs) of virulent and less-virulent strains of the periodontal pathogen *P. gingivalis* differ by more than 20% [135], and it has been estimated that the pan genome of the oral bacterium *Streptococcus agalactiae* would still be insufficiently characterized even if the full genomes of more than a hundred strains were sequenced [136]. Thus polymorphic species, which are typical of microbiome endemic pathogens (e.g. *Helicobacter pylori* [137], *Neisseria meningitidis* [136] and *P. gingivalis* [135]), pose greater genome reconstruction challenges than epidemic monomorphic pathogens (e.g. *M. tuberculosis*, *M. leprae* and *Y. pestis*), which are largely clonal [2] and may be more easily scaffolded onto modern reference genomes. The future of ancient microbiome studies will require the development of novel genome assembly techniques and algorithms.

#### (iii) Shotgun metaproteomics

Shotgun metaproteomics is a new tool in microbiome studies that allows both microbial and host proteins within the microbiome to be characterized simultaneously. An advantage of shotgun metaproteomics compared with metagenomics is that rather than being limited to the genetic content of a bacterial community, which represents the blueprint of functional potential, shotgun metaproteomics provides direct access to actual protein functions being performed [138,139]. This can be especially useful for examining pathogen–host interactions and immune response [140,141], as has been recently demonstrated in studies of mummified soft tissue [142] and ancient dental calculus [39]. As an emerging technique, shotgun metaproteomics faces important challenges, including analysis bottlenecks with respect to sample throughput, standardization, replicability and the establishment of appropriate reference databases. Many of these challenges are shared with shotgun metagenomics, but others are unique to protein analysis. For example, proteins deriving from a common DNA sequence can appear in alternative isoforms and exhibit different post-translational modifications that are difficult to predict based on the genome sequence alone and instead must be empirically tested and validated. Additionally, protein sequencing is less straightforward than DNA sequencing, and sequence interpretation relies heavily on spectra comparison to reference databases that, by necessity, are often limited in scope or size in order to reduce computational complexity. However, despite these challenges, shotgun metaproteomics is a rapidly developing and growing field that promises to yield unique insights into the role of host microbiota in ancient health and disease [116].

### (d) Authentication and contamination

In addition to standard ancient DNA contamination precautions [27,28], the investigation of ancient microbiomes requires several further considerations. Because bacteria, rather than host DNA, are the organisms of interest, identifying sources of contamination becomes a leading challenge. Bacteria are ubiquitous, and contamination can originate from myriad sources, most notably the burial environment, post-excavation handling and the laboratory. Even the air around us contains more than a thousand bacterial species [22,143], many of which may be shed from our own bodies [144]. The analysis of ancient microbial DNA has been considered problematic by some because of the difficulties of eliminating contamination from modern sources; however, in practice there are many measures that can be taken to assess authenticity and reduce contamination artefacts. Specifically, with respect to metagenomic community-level characterization of ancient microbiomes, there are three principal challenges: (i) post-mortem community alterations (decomposition or modern contamination) that can alter bacterial diversity estimates and skew community structure, (ii) DNA damage artefacts that can artificially inflate bacterial diversity estimates, and (iii) laboratory reagent and sample crossover contamination.

#### (i) Decomposition and environmental contamination

Post-mortem microbial community alterations due to *in situ* decomposition and/or environmental contamination are particularly challenging to ancient microbiome studies, as they can artificially inflate or reduce bacterial diversity, as well as skew community structure. The bioinformatics tool SourceTracker [32] has been shown to be both highly sensitive and effective at detecting decomposition and exogenous contamination in ancient microbiome samples [39,145]. Using this tool, ancient microbiome samples can be tested for potential contaminants using published datasets (e.g. skin microbiome, compost and soil) and/or locally generated datasets (e.g. laboratory air samples, and bone or dentine samples as a proxy for infiltrated soil bacteria). Although at present it is not yet possible to use this tool to subtract identified contamination from sample datasets, it is nevertheless an objective and effective screening tool for identifying authentic ancient microbiome samples.

#### (ii) Damage artefacts

Another challenge in ancient microbiome community characterization is damage artefacts. Cytosine deamination and other miscoding lesions are characteristic of ancient DNA, and they are even used to detect and authenticate genuine ancient DNA sequences [146]. With sufficient depth of coverage obtained through cloning or NGS deep sequencing, these miscoding lesions can be identified and removed from conventional ancient DNA datasets, but they pose a major challenge in metagenomic analyses of microbial communities, where a single nucleotide change could represent either a damage artefact or a novel organism. Microbiomes typically contain thousands of taxa at frequencies that differ by orders of magnitude. Therefore, the depth of coverage for all but the most abundant taxa is expected to be very low, and sequence alignment cannot be used to distinguish damage from true sequence differences. In order to reduce artificial inflation of bacterial diversity due to damage, a high fidelity damage-sensitive DNA polymerase, such as Phusion Hot Start II (Thermo Scientific), can be used for NGS library generation [39]. Although damage-based ancient DNA authentication tools, such as mapDamage [146], may be incompatible with this approach, other ancient DNA authentication methods, such as testing for asymmetrical molecular behaviour on the basis of ancient DNA fragment length [147], can still be applied.

#### (iii) Laboratory reagent and sample crossover contamination

Low-level contamination of laboratory reagents, especially primers and dNTPs, can pose serious challenges when using universal bacterial primers. Fortunately, new protocols for reagent decontamination using a heat labile double stranded DNase [26] are highly effective, and when used consistently, these protocols largely eliminate reagent contamination as a major concern in current ancient DNA research. Moreover, because the majority of ancient microbiome DNA is bacterial in origin, amplicon-based approaches using universal bacterial primers typically require only moderate PCR cycling (30–35 cycles), again reducing reagent contamination risk.

Finally, sample crossover contamination at commercial NGS sequencing facilities can introduce foreign DNA sequences into a dataset, and for this reason it is strongly recommended to index ancient DNA libraries with short, sample-specific barcodes prior to sequencing [148]. While commercial NGS library kits offer this indexing ability, it is important to consider that many other laboratories use these same kits, and so to reduce crossover contamination more effectively it may be preferable instead to custom order unique, or at least less common, barcode sequences.

## 6. Conclusion

We have entered a new era in palaeomicrobiology. NGS has allowed the recovery of major epidemic pathogens and elucidated the causes of historic pandemics and specific palaeopathologies. At the same time, major international initiatives to investigate the human microbiome have revealed both the importance of human-associated microbes in basic human life functions, as well as their role in a variety of acute and chronic diseases. Recent NGS-based palaeomicrobiology studies have revealed dental calculus to be an important reservoir of ancient human oral microbiomes, offering a unique opportunity to examine the links between human health, diet, lifestyle and the environment throughout the course of human evolution. Although still in its infancy, microbiome palaeomicrobiology has great potential to elucidate the dynamic and intimate relationship between humans and their microbes and to lead to a deeper understanding of the place of our ancient microbial self in the modern world.
